# New Insights into *Capsicum* spp Relatedness and the Diversification Process of *Capsicum annuum* in Spain

**DOI:** 10.1371/journal.pone.0116276

**Published:** 2014-12-29

**Authors:** Susana González-Pérez, Ana Garcés-Claver, Cristina Mallor, Luis E. Sáenz de Miera, Oreto Fayos, Federico Pomar, Fuencisla Merino, Cristina Silvar

**Affiliations:** 1 Department of Ecology, Plant and Animal Biology, University of Coruña, A Coruña, Spain; 2 Agrifood Research and Technology Centre of Aragón (CITA), Zaragoza, Spain; 3 Departament of Molecular Biology, University of León, León, Spain; Huazhong University of Science and Technology, China

## Abstract

The successful exploitation of germplasm banks, harbouring plant genetic resources indispensable for plant breeding, will depend on our ability to characterize their genetic diversity. The Vegetable Germplasm Bank of Zaragoza (BGHZ) (Spain) holds an important *Capsicum annuum* collection, where most of the Spanish pepper variability is represented, as well as several accessions of other domesticated and non-domesticated *Capsicum* spp from all over the five continents. In the present work, a total of 51 *C. annuum* landraces (mainly from Spain) and 51 accessions from nine *Capsicum* species maintained at the BGHZ were evaluated using 39 microsatellite (SSR) markers spanning the whole genome. The 39 polymorphic markers allowed the detection of 381 alleles, with an average of 9.8 alleles per locus. A sizeable proportion of alleles (41.2%) were recorded as specific alleles and the majority of these were present at very low frequencies (rare alleles). Multivariate and model-based analyses partitioned the collection in seven clusters comprising the ten different *Capsicum* spp analysed: *C. annuum*, *C. chinense*, *C. frutescens*, *C. pubescens*, *C. bacatum*, *C. chacoense* and *C. eximium*. The data clearly showed the close relationships between *C. chinense* and *C. frutescens*. *C. cardenasii* and *C. eximium* were indistinguishable as a single, morphologically variable species. Moreover, *C. chacoense* was placed between *C. baccatum* and *C. pubescens* complexes. The *C. annuum* group was structured into three main clusters, mostly according to the pepper fruit shape, size and potential pungency. Results suggest that the diversification of *C. annuum* in Spain may occur from a rather limited gene pool, still represented by few landraces with ancestral traits. This ancient population would suffer from local selection at the distinct geographical regions of Spain, giving way to pungent and elongated fruited peppers in the South and Center, while sweet blocky and triangular types in Northern Spain.

## Introduction

The genus *Capsicum* belongs to the *Solanaceae* family and have its origins in the tropical South American region centered in what is now Bolivia [1], [2]. Currently, the number of recognized species in the genus is twenty-seven, five of which were domesticated from distinct events at different primary diversification centers [3], [4]. These five cultivated forms of *Capsicum*: *C. annuum* L., *C. chinense* Jacq., *C. frutescens* L., *C. baccatum* L. and *C. pubescens* Ruiz et Pav., represent some of the most economically important vegetable crops worldwide due to their versatile and innovative food and non-food uses [5]. These species are thought to have been domesticated independently in at least two regions of the New World; *C. annuum* and *C. frutescens* in Mesoamerica and *C. chinense*, *C. baccatum* and *C. pubescens* in South America [6]. *Capsicum* species have been divided into three complexes based on cytogenetics and cross fertility. The *C. annuum* complex contains *C. annuum*, *C. chinense*, *C. frutescens*, their wild relatives and *C. galapagoense* Hunziker. These species are integrated into a morphological continuum and they are potentially crossable with ease, although compatibility might be affected by the pollinated genotypes, the direction of crossing or even by the reciprocal 1/8 chromosome translocation event that differentiates *C. annuum* from its related species [7]–[9]. Many authors have argued that at least *C. frutescens* and *C. chinense* should be combined into one species [10], [11]. The *C. baccatum* complex comprises *C. baccatum*, *C. praetermissum* Heiser et Smith and *C. tovarii* Eshbaugh, Smith et Nickrent, although the location of the last wild species is still disputable [11]–[13]. Finally, the *C. pubescens* complex is constituted by *C. pubescens*, *C. cardenasii* Heiser et Smith and *C. eximium* Hunziker. Even though the last two complexes are sexually isolated from each other and from the first one, viable hybrids have been obtained between *C. annuum* and *C. baccatum* and different methodologies have been proposed to cross *C. pubescens* and *C. annuum* [14], [15]. The wild species *C. chacoense* Hunziker has not been clearly assigned to one of these genetic pools, and it has been considered to be in either the *C. baccatum* or the *C. annuum* complex [11]–[13].

Since its domestication in pre-Columbian times, peppers have migrated worldwide. They were introduced into Europe at the end of the 15^th^ century. Later on they were dispersed to Mediterranean countries, and afterwards to Africa, India and China [3], [16]. *C. annuum* was the most successful in this conquest, probably due to it being the first *Capsicum* that arrived to Europe, rather than to any superior agronomic trait [17]. *C. chinense* and *C. frutescens* became also popular in Africa and Asia, whereas *C. baccatum* and *C. pubescens* mostly remained in South America and Andean regions [16]. In these secondary diversification centers, the different species were selected by farmers over centuries of cultivation to fit the divergent agro-climatic environments of specific regions, giving rise to local lines or landraces and resulting in the great phenotypic diversity of pepper cultivars found nowadays [18], [19]. Among the five domesticates, *C. annuum* is the most widespread and economically important *Capsicum* species worldwide as well as the most used in commercial cultivar breeding programs. *C. annuum* was likely domesticated in Mexico from the wild bird pepper or “Chiltepin” (*C. annuum* var. *glabriusculum*) [1], [20], [21]. The fruit of the wild progenitor is small, erect, red-coloured, pungent, deciduous and soft-fleshed. Domestication and subsequent steps of artificial selections led to the great variation in size, form, colour and pungency of contemporary *C. annuum* fruits, depending on human preferences at the different diversification territories [22]. In general, continued selection was driven to obtain lines with non-deciduous, pendant, larger and non-pungent fruits with greater shape variation and increased fruit mass [23]. Nowadays, *C. annuum* contains both small pungent peppers, mainly used for spice and condiments, and large-fruited blocky (Bell) types, which are the most economically important around the world [16]. In the last century, modern plant breeding provoked that traditional landraces gave way to commercial varieties and hybrids with higher, more uniform yields and frequently carrying resistances to diseases [24]. As a consequence, modern and highly performing cultivars have pragmatically replaced the diversified and heterogeneous landraces all around the world. This phenomenon led to a considerable reduction of the genetic diversity of pepper; threatening their cultivation with genetic erosion [25], as these genetically uniform cultivars possess a higher propensity to become more susceptible to biotic and abiotic stresses inherent to the crop [26]. Broadening the genetic basis of this and other economically important crops represent a challenge in order to respond to climate change and increasing global food demand in the successive decades [27]. Landraces, wild ancestors and related species are important reservoirs of genetic variation that have not been always exploited in breeding programmes. Since the early 20^th^ century, great efforts have been made by private, national and international initiatives to preserve the variability associated with each crop through the maintenance of germplasm collections [28]. In Spain, as in other parts of Southern Europe, the versatility of agro-climatic regions and the heterogeneity of the land favoured the survival in cultivation of a large number of specifically adapted landraces very diverse phenotypically. The majority of these landraces are conserved at the Vegetable Germplasm Bank of Zaragoza (BGHZ) (Zaragoza, Spain), which also contains several accessions of other domesticated and non-domesticated *Capsicum* spp from all over the five continents [29].

The evaluation of *Capsicum* spp genetic resources is essential to investigate the relationships among the different species and to outline the presumed diversification processes of each at the different secondary diversification centers. The characterization of these resources will be also compulsory to identify valuable accessions for breeding programmes and to develop efficient conservation strategies not only in genebanks but also *in situ*. Several methodologies to analyse genetic variability, such as phenotypic descriptors and molecular markers, have been widely used in crop diversity studies. However, the latter methods (principally those based on PCR) became of preference because they are not under the influence of environmental conditions or plant development factors [30]. Different types of DNA markers, such as RFLPs, RAPDs, AFLPs or microsatellite (SSRs), have been developed and used in peppers to determine the relationships and levels of genetic variation in wild and domesticated *Capsicum* spp [31]–[34]. Over the last decade, SSR markers have emerged as the most widely-used genotyping markers in plants because of their co-dominance, stability, capacity of multi-allelic detection, ease of application and excellent sensitivity, particularly between species [30]. Recently, several groups of pepper microsatellites, both genomic and EST-based markers, came to be available for diversity studies and many of them have been mapped in intra- and interspecific populations [35]–[42]. Such markers allow now a better understanding of the extent and patterns of genetic diversity within and among worldwide *Capsicum* germsplasm collections and pave the way towards marker-trait association studies based not only in mapping populations but also in association mapping approaches.

The main goal of the present work was to assess the genetic diversity and structure present in the *Capsicum* genetic resources maintained at the BGHZ by using a large set of microsatellite markers, as well as to investigate the relatedness among the representatives of the different *Capsicum* spp., emphasizing in the gene pool composed by *C. annuum* accessions originate in Spain.

## Materials and Methods

### Plant Material

One hundred and two accessions were selected from the pepper (*Capsicum* spp) germplasm collection maintained at the Vegetable Germplasm Bank of Zaragoza (BGHZ) (Zaragoza, Spain) (Table S1). The majority of accessions belong to the domesticated species *C. annuum* (51), *C. chinense* (14), *C. bacatum* (11), *C. frutescens* (8) and *C. pubescens* (5). *C. annuum* accessions are mainly landraces from Spain. The other fifteen accessions represent five closely related wild species (*C. chacoense*, *C. eximium*, *C. galapagoense*, *C. cardenasii* and *C. tovarii*) (Table S1). All accessions were previously classified according to morphological traits [29], based on the IPGRI descriptors for *Capsicum* [43]. Fruit shapes were also coded according to IPGRI and, when possible, the accessions were categorized into the horticultural types defined by Bosland [16], [44].

### DNA extraction and microsatellite genotyping

Genomic DNA was isolated from young leaves of each accession using the CTAB method [45] and stored at −20°C until used. Fifty five publicly available microsatellite markers, uniformly distributed across the twelve pepper chromosomes, were mostly selected according to their broad transferability and high polymorphism in different *Capsicum* spp [34], [37], [38], [46] (Table S2). Additional information on the markers positions in chromosomes were retrieved from Barchi et al. [47], Wu et al. [39], Mimura et al. [41], Sugita et al. [42], Nicolai et al. [48], and through the SOL Genomics Network (http://solgenomics.net/). The putative presence of the pungency trait in the *Capsicum* accessions was assessed by using the molecular marker MAP1, which was developed based on a DNA sequence with high similarity to *Pun1* locus [49]. PCR amplification and detection of microsatellite markers were performed according to the methodology reported by Schuelke [50] based on the fluorescently labelling of universal primers. Briefly, an M13 tail (5′-CACGACGTTGTAAAACGAC-3′) was added to the 5′ end of each publicly available forward primer cited above. A universal primer with a complementary sequence to the M13 tail was labelled with different fluorophores (6-Fam, Hex or Ned). PCR was carried out in a final volume of 15 µl, which contained 50–75 ng of genomic DNA, 1× PCR Buffer (NZYTech), 2.5 mM MgCl2 (NZYTech), 0.02 µM of the forward primer, 0.2 µM of reverse primer, 0.18 µM of the universal primer, dNTPs (Fermentas) at 0.2 mM each, and 0.4 U of Taq DNA Polymerase (NZYTech). All fragments were amplified using the following touchdown PCR profile: an initial denaturing step of 5 min at 94°C was followed by 35 cycles with denaturation at 94°C for 30 s and extension at 72°C for 30 s, respectively. The annealing temperature was decreased in 0.5°C increments from 62°C in the first cycle to 56°C and was then kept constant for the remaining 35 cycles (always 30–50 s). A final extension step was performed at 72°C for 10 min. DNA fragments were resolved in an Applied Biosystems 3130xl Genetic Analyzer. The allele sizes were assigned with the GeneMapper 3.7 software (Applied Biosystems).

### Data analyses

The number of observed alleles per locus, the observed heterozygosity (H_o_ = number of heterozygous individuals/number of individuals scored) and the Nei's unbiased gene diversity index (uH_e_) [51] were calculated using GenAlex software v6.5 [52]. PIC (Polymorphic Information Content) values for each marker were computed according to the formula of Botstein et al. [53] implemented in the Excel Microsatellite Toolkit [54].

The marker data were used to generate a 0/1 matrix (presence/absence of allele at the marker locus) that was employed to estimate the genotypic distances between accessions. Pairwise similarities were calculated using the Dice coefficient [55]. A tree depicting relationships of the germplasm was built from the distance matrix using the Neighbour-Joining (NJ) method (1000 bootstraped). Diversity within the collection was further assessed using principal coordinates analysis (PCoA). All these analyses were carried out using R software (v3.0.2) [56]. The graphical representation of the tree was performed with MEGA software v6 [57].

In order to investigate the population structure and to assign individuals to populations based on the SSR genotypes, a Bayesian model-based clustering procedure implemented in the software STRUCTURE 2.3.4 was used [58]. The tests were performed using an admixture model with correlated allele frequencies and by setting the number of populations (*K*) from 1 to 10, with 10 independent simulations for each *K*. Each run consisted of a burn-in period of 500,000 steps and 10^6^ Markov Chain Monte Carlo (MCMC) repetitions. In order to assess the best *K* value supported by the data, the Δ*K* method described by Evanno et al. [59] was used through Structure Harvester v. 6.93 [60] to examine the rate of change in successive posterior probabilities over the range of *K* values. When the results suggested that the *K* groups could be further structured in sub-groups, a second-level of STRUCTURE analysis was performed individually for each *K* group [61]. Genotypes were assigned to the group (or sub-group) for whom they had the highest membership coefficient, considering strong affinity when the membership coefficient (*qI*) was ≥80% [62], [63]. CLUMPP [64] was employed to generate Q values for groups or sub-groups from the STRUCTURE data using the Greedy *K* algorithm.

## Results

### Marker summary and genetic diversity

Eight loci (AA840692, GPMS113, CAMS864, HpmsE004, Hpms1-214, GP20087, GPMS008 and HpmsE128) did not amplify in any accession. The primer Hpms2-21 produced only one amplicon in the *C. annuum* group and the SSR Hpms1-111 generated multiple bands on the agarose gels. Loci CAMS647, GP20068, Hpms1-172 and GPMS117 did not exhibit easily interpreted electropherograms in the genetic analyser. All these markers were not considered in subsequent analyses. Among the remaining 41 informative loci, two (GPMS171 and GPMS164) showed one allele with a frequency close to 95%, and they were excluded from all analyses to avoid potential errors in estimating genetic diversity. A few markers did not deliver any amplicon in some species and they were considered as null alleles: particularly *C. tovarii* and *C. cardenasii* (Hpms1-5, HpmsE120, CA523558, Hpms2-45, GPMS178, CA514272, EMPS342, HpmsE082, Hpms1-3), *C. pubescens* (Hpms1-165 and Hpms1-5), *C. eximium* (EPMS342 and HpmsE125) and *C. chacoense* (GPMS159) (Table S3). An analysis per group revealed that various markers resulted monomorphic in some species. Thus, *C. frutescens* showed the highest rate of polymorphism (97.4%), followed by *C. baccatum* (89.7%), *C. chacoense* (89.5%), *C. annuum*/*C. chinense* (84.6%) and *C. pubescens/C. eximium* (58.9%) (Table S3).

The 39 SSR loci amplified a total of 381 alleles in the 102 accessions analysed, ranging from 3 (HpmsE120 and CP10023) to 23 (Hpms1-5), with an average of 9.8 distinct alleles per locus. Allele size oscillated between 118 and 379 bp. The total number of alleles highly varied among the *Capsicum* spp. The highest number of alleles was found in *C. annuum* (157), *C. chinense* (125) and *C. frutescens* (124), whereas the lowest was detected for *C. pubescens* (70), *C. galapagoense* (68) and *C. eximium* (61) (Table S3). A sizeable proportion of alleles (41.2%) was recorded as unique alleles, i.e., alleles present in only one species and absent in the others. Although differences in sample size need to be considered, the number of unique alleles was much higher in *C. annuum*, *C. chacoense* and *C. chinense* (46, 29 and 24 unique alleles, respectively), whereas these alleles were absent in *C. galapagoense*. *C. tovarii* presented eight unique alleles, despite to be represented by only one accession (Table S3). The majority of these were present at very low frequencies (rare alleles) in *C. annuum* (58.7%), but not in *C. chacoense* or *C. chinense*, where specific alleles were found in more than 5% of the individuals of the corresponding group. The polymorphic information content (PIC), which depicts the number of alleles and their distribution, was calculated to determine the interspecific and intraspecific informativeness of each marker. Considering the whole investigated set of *Capsicum* accessions, the PIC value ranged from 0.88 (Hpms1-5) to 0.13 (CP10023) with an average of 0.66. The most informative markers varied among the different species (Table S4).

The observed heterozygosity (H_0_) was very low for all markers with a mean of 0.074 for the whole set. The Nei's unbiased gene diversity index ranged from 0.13 (CP10023) to 0.89 (Hpms1-5), with an average of 0.69 for all accessions (Table 1). The mean SSR diversity parameters were considered in the different *Capsicum* species including more than two accessions (Table 1). The mean number of alleles per locus varied from 1.80 (*C. pubescens*) to 4.03 (*C. annuum*). However, this parameter is not very meaningful, as it is biased due to the unbalanced number of accessions between species. The observed heterozygosity and Nei's index, which summarize the fundamental genetic variation of a group, should be more appropriate. In this manner, the lowest H_o_ was observed for *C. annuum* (0.03), while the highest was detected for *C. pubescens* (0.15). *C. frutescens* was the most diverse, while the least was *C. pubescens* (average diversity index across all loci of 0.54 versus 0.28) (Table 1).

**Table 1:** Diversity parameters in the different *Capsicum* species and in the entire collection.

Group	N	Na	Ho	uHe
*C. annuum*	51	4.03	0.03	0.42
*C. chinense*	14	3.20	0.12	0.44
*C. baccatum*	11	3.00	0.12	0.43
*C. frutescens*	8	3.13	0.10	0.54
*C. pubescens*	5	1.80	0.15	0.28
*C. chacoense*	7	3.00	0.11	0.49
Total	96	9.76	0.07	0.70

Species with less than two accessions are not represented. N = number of accessions, Na = average number of alleles per locus, Ho = observed heterozigosity, uHe = Nei's unbiased gene diversity index.

The comparison of non-Spanish and Spanish *C. annuum* accessions was based on 29 polymorphic loci that generated a total of 143 alleles with an average of 4.9 alleles per locus. Thirty-seven alleles appeared only in the Spanish group and almost half of these (40.5%) were considered rare alleles. The diversity index across all loci was smaller for the Spanish group (0.53) than for the non-Spanish *C. annuum* (0.65) (data not shown).

### Multivariate analysis

**Figure 1:**
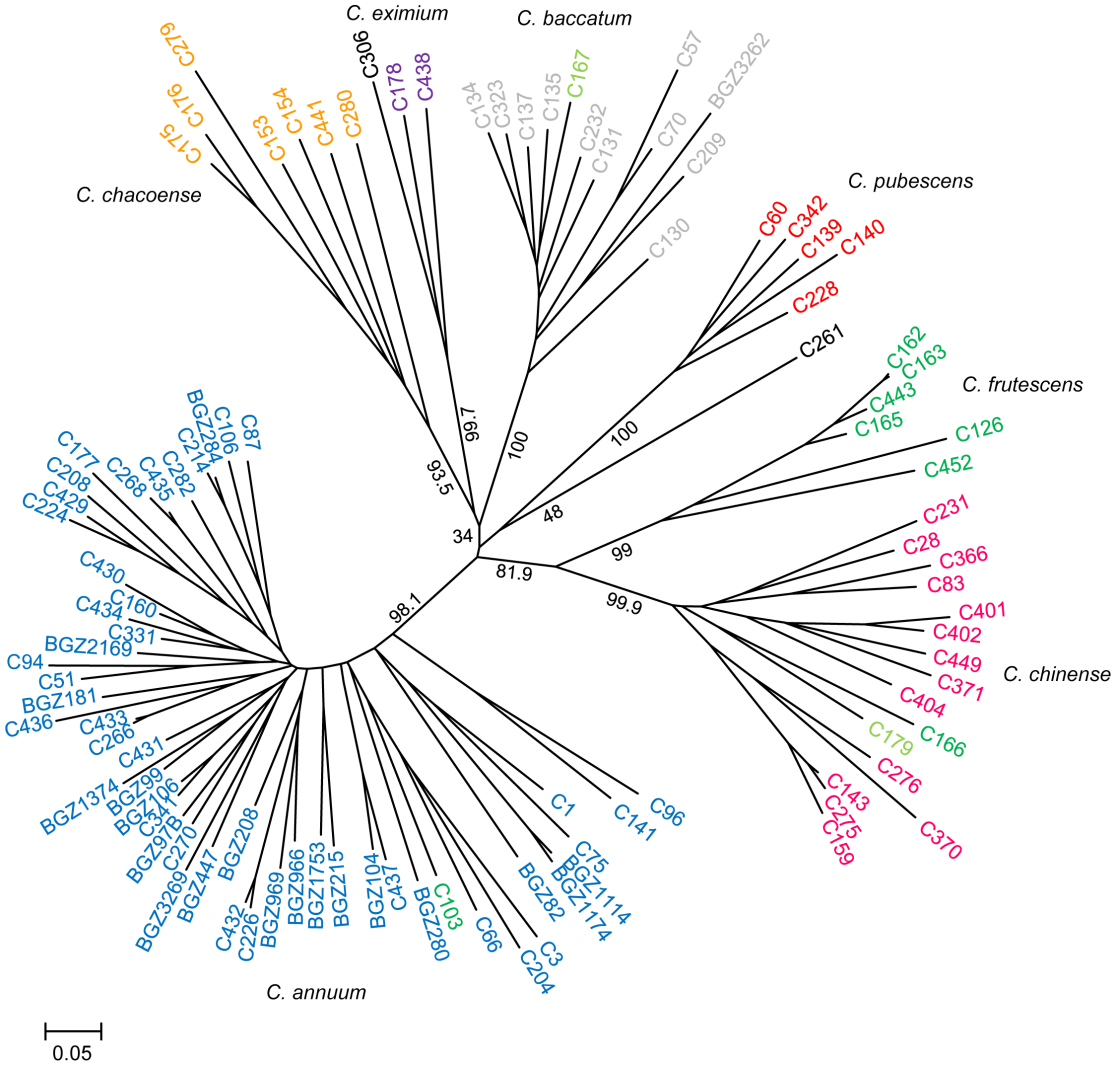
NJ tree of 102 *Capsicum* accessions based on 39 SSR markers. Only bootstrap values that supported each species or complex have been printed. Numbers are the percentage of bootstrap support. *C. cardenasii* C306 and *C. tovarii* C261 are represented in black. *C. galapagoense* accessions C167 and C179 are shown in light green.

Pairwise similarities among accessions were computed using the Dice coefficient and the resulting matrix was employed to generate a dendrogram using the Neighbor-Joining clustering algorithm. The NJ tree was well resolved and showed well-supported groups corresponding to the different species (Fig. 1). Only few displacements were observed, some accessions not being grouped into clusters that corresponded to their morphological classification. Thus, the *C. frutescens* accession C103 clustered with the *C. annuun* group, although with a lower value of bootstrap (40.5%). In the same manner, the accession C166 (*C. frutescens*) was included in the *C. chinense* branch (bootstrap 41.2%).

The cultivated *C. annuum* accessions were clustered together in a single group, with a bootstrap of 98.1%. The other domesticated species *C. chinense*, *C. frutescens*, *C. pubescens* and *C. baccatum* were clearly into distinct branches supported by high values of bootstrap (>80%). The *C. chinense* accessions were visibly separated from *C. frutescens* accessions, but both groups were clustered tightly with high bootstrap support (81.9%). *C. pubescens* accessions were clustered with 100% of confidence and closely related to *C. tovarii*, although with low bootstrap support (ca. 48%). *C. baccatum* accessions were in a cluster supported by a 100% bootstrap value and they seemed related to the wild species *C. eximium*, *C. cardenasii* and *C. chacoense*, although under a very low bootstrap-supported node (ca. 34%). *C. eximium* and *C. cardenasii* were clustered together with a bootstrap value of 99.7%. The wild species *C. chacoense* formed a distinct group supported by a 93.5% bootstrap, being more related to *C. eximium* and *C. baccatum* (Fig. 1). *C. galapagoense* C167 and C179 clustered with the *C. baccatum* and *C. chinense* groups, supported by bootstrap values of 45.9% and 41.2%, respectively (Fig. 1).

**Figure 2:**
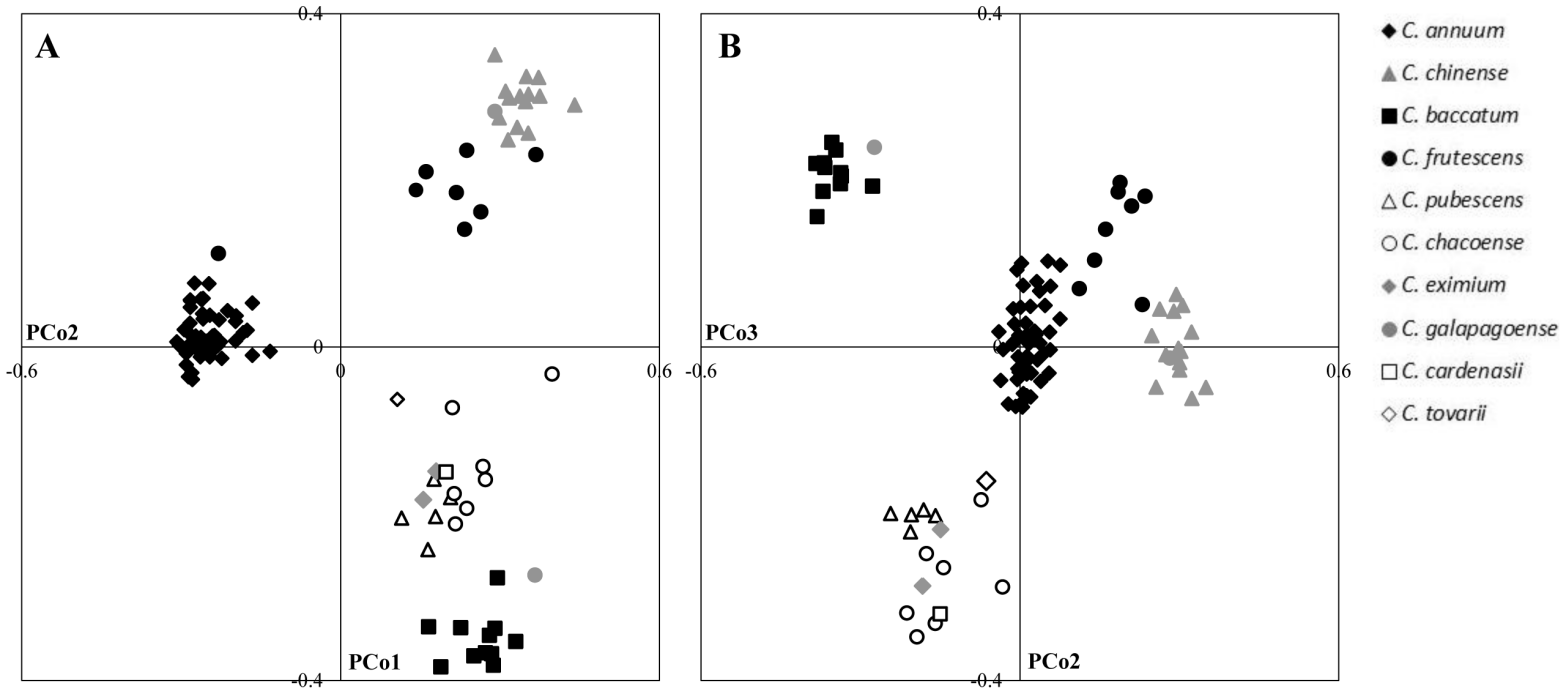
Principal Coordinate Analysis (PCoA) of 102 *Capsicum* accessions based on 39 SSR markers. A) Coordinate 1 (PCo1) vs Coordinate 2 (PCo2). B) PCo2 vs PCo3.

Genetic relationships among *Capsicum* ssp was further investigated using principal coordinate analysis (Fig. 2). The eigenvalues revealed that the first three principal coordinates accounted for 31.9% of the total genetic variability. The projection of accessions in the three-dimensional plane basically agreed with that of the NJ tree. The first axis alone explained 17.9% of the variance, and clearly separated the *C. annuum* accessions from the other species (Fig. 2a). The second and third coordinates explained 8.9% and 5.0% of the marker variance, respectively, and split the three established *Capsicum* complexes; *C. annuum* complex (*C. annuum*, *C. frutescens* and *C. chinense*), the *C. baccatum* complex (*C. baccatum*) and the *C. pubescens* complex (*C. pubescens*, *C. eximium* and *C. cardenasii*) (Fig. 2b). As deduced from the hierarchical clustering, *C. tovarii* was located close to the *C. pubescens* complex, while *C. galapagoense* accessions C167 and C179 were grouped with *C. baccatum* and *C. chinense*, respectively. Interestingly, *C. chacoense* accessions did not form a specific group and they were clustered together with the *C. pubescens* complex, differing from the NJ tree results.

### Genetic structure of the collection

**Figure 3:**
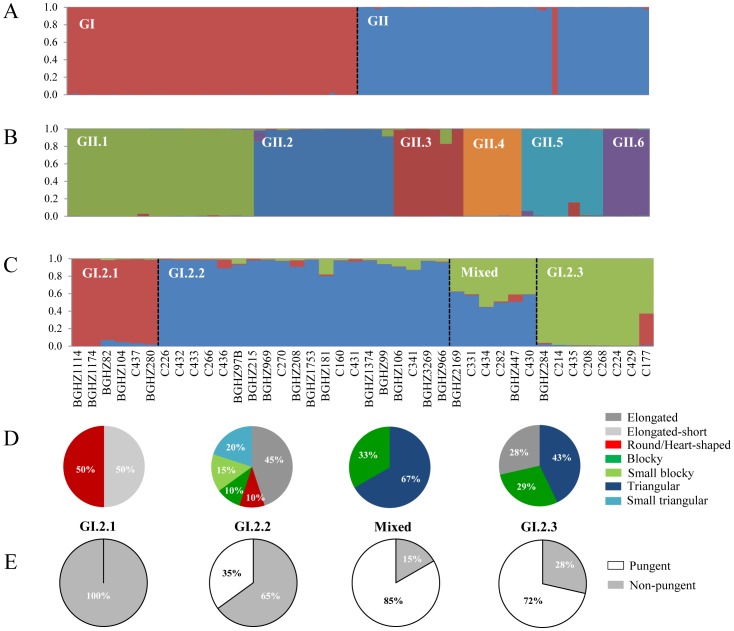
Bayesian clustering for *Capsicum* accessions genotyped at 39 loci. A) Structure bar plots based on 102 *Capsicum* accessions at *K* = 2, GI indicates the *C. annuum* accessions and GII the non-*annuum* group. B) Structure bar plots based on 50 *Capsicum* non-*annuum* accessions at *K* = 6, GII.1 = *C. chinense*, GII.2 = *C. baccatum*, GII.3 = *C. frutescens*, GII.4 = *C. pubescens*, GII.5 = *C. chacoense* and GII.6 = *C. eximium, C. cardenasii* and *C. tovarii*. C) Spanish *C. annuum* population sub-structure for *K* = 3. D) Distribution of the different fruit morphologies in the groups defined by Structure within the Spanish *C. annuum* panel. E) Presence (named as “pungent”) or absence (“non-pungent”) of the MAP1 allele for pungency in each group established by the model based analysis.

The software STRUCTURE was applied to define the population structure and to assign individuals to populations. In the first analysis, the log probability of data (ln[Pr(*X*/*K*)]), after the Evanno et al. [59] correction, showed a clear maximum for Δ*K* = 2 (S1A Fig.). This partitioning level corresponds to a very strong differentiation in two major groups, one corresponding to the *C. annuum* accessions (GI) and a second one which include the other species (GII), all of them with membership coefficients *qI*>80% (Fig. 3a). A more exhaustive exploration of the Δ*K* results obtained after analysis with Structure Harvester revealed a smaller peak at *K* = 8, suggesting that the two major groups GI and GII could be sub-structured. Therefore, each of them was submitted separately to further STRUCTURE analysis. The subsequent evaluation of the Δ*K* statistic indicated that *K* = 6 was the most probable number of clearly differentiated genetic sub-groups of *Capsicum* spp. within the non-*annuum* group (S1B Fig., Fig. 3b). The clustering of the different species was very similar to the results for both the hierarchical clustering and discriminant analysis, since all sub-groups displayed a clear cut structure with no or very few admixture. The first sub-group (GII.1) included the *C. chinense* accessions. The second (GII.2) corresponded to *C. baccatum*, while the third sub-group (GII.3) comprised the *C. frutescens* accessions. The fourth one (GII.4) included *C. pubescens* whereas all the *C. chacoense* accessions were assigned to the fifth sub-group (GII.5). The sixth cluster (G-II.6) consists of *C. eximium* together with *C. cardenasii* and *C. tovarii*. *C. galapagoense* accessions C167 and C179 were resolved in *C. baccatum* and *C. chinense*, respectively (Fig. 3b).

A Bayesian analysis was also performed individually for the *C. annuum* group (GI). In this case, the highest Δ*K* value matched to *K* = 2 (S1C Fig.), which mainly corresponded to the differentiation between non-Spanish *C. annuum* accessions (GI.1) and accessions from Spain (GI.2) (S2A Fig.). Some Spanish accessions assembled with the non-Spanish group at coefficients *qI*>95%, while few of them showed admixture, with half of their genome belonging to two different groups (S2A Fig.). The lack of stabilization of the ln[Pr(*X*/*K*)] across runs at *K* = 2 and the presence of secondary smaller peaks at increasing *K*s, pointed at some underlying sub-structure within the group of *C. annuum* genotypes. The independent analysis of the Spanish group showed a maximum for *K* = 3 (S1D Fig., Fig. 3c). The sub-groups GI.2.1 and GI.2.2 included 6 and 20 genotypes with more than 80% of membership into each cluster, respectively. The sub-group GI.2.3 comprised 7 genotypes with *qI*>0.8. There were six accessions that showed less than 0.60 memberships in any given sub-group, but they shared similar *qI* for both the GI.2.2 and GI.2.3 clusters, indicating a mixed sub-group comprised by a common overall genomic constitution (Fig. 3c). The genetic relationships among all sub-groups showed rather good coherence with pepper fruit size and shape and partially with the geographical origin of accessions. The IPGRI descriptor for overall pepper fruit shape comprises mostly five categories: campanulate, almost round, elongate, blocky and triangular [43]. On the basis of STRUCTURE analysis, we have subdivided the latter sections into small and large types. The sub-group GI.2.1 was characterized by accessions with small size fruits and shapes from elongated short (50%) (between ‘Piquin’ and ‘Serrano’ types, according to Bosland [16], [44]) to almost round and heart-shaped (similar to ‘Cherry’ and ‘Pimiento’ types) (50%). This group is also defined by taller plants (average >100 cm) coming from the Canary Islands and a small region in the centre of Spain (Fig. 3d). The second sub-group (GI.2.2) included mostly accessions with slightly shorter plants (around 90 cm) carrying fruits with elongated morphology (‘Cayenne’ type) (45%) and small blocky and small triangular types (30%). The majority of these accessions are from the Center and South of Spain, but a reduced number, comprising the small blocky and triangular shapes (C226, C432, C433, C266 and C436), come from the North. The third sub-group (GI.2.3) comprised accessions with smaller plants (<75 cm) bearing principally triangular (similar to ‘Ancho’ and ‘Anaheim’ types), and blocky (‘Bell’ type) fruits of larger sizes (72%). These accessions are mainly originally from Northern Spain. The Mixed group was composed by triangular and blocky types also originated in the North (Fig. 3d). The potential presence of the pungency trait in the Spanish accessions was tested with the molecular marker MAP1. All accessions in the sub-group GI.2.1 carried an allele associated to pungency in the MAP1 marker (Fig. 3e). The specific fragment for pungency was also recorded in sixty percent of accessions in sub-group GII.2.2, mainly corresponding to the fruits with elongated shape. Regarding sub-group GI.2.3 and Mixed group, the majority of accessions (71.4% and 83.3%, respectively) displayed the non-pungent allele (Fig. 3e). Considering that the increase of the log did not taper off for *K* = 3, the clustering of accessions obtained at higher *K* values was also examined. When increasing *K*, the GI.2.3 group remained almost inseparable (S2B Fig.). The GI.2.1 and GI.2.2 groups became divided into smaller subgroups, with some accessions showing now a mixed membership. Interestingly, the group formed by small blocky and triangular types from the North in cluster GI.2.2 set apart at *K* = 4 and more clearly at *K* = 5 (S2B Fig.).

**Figure 4:**
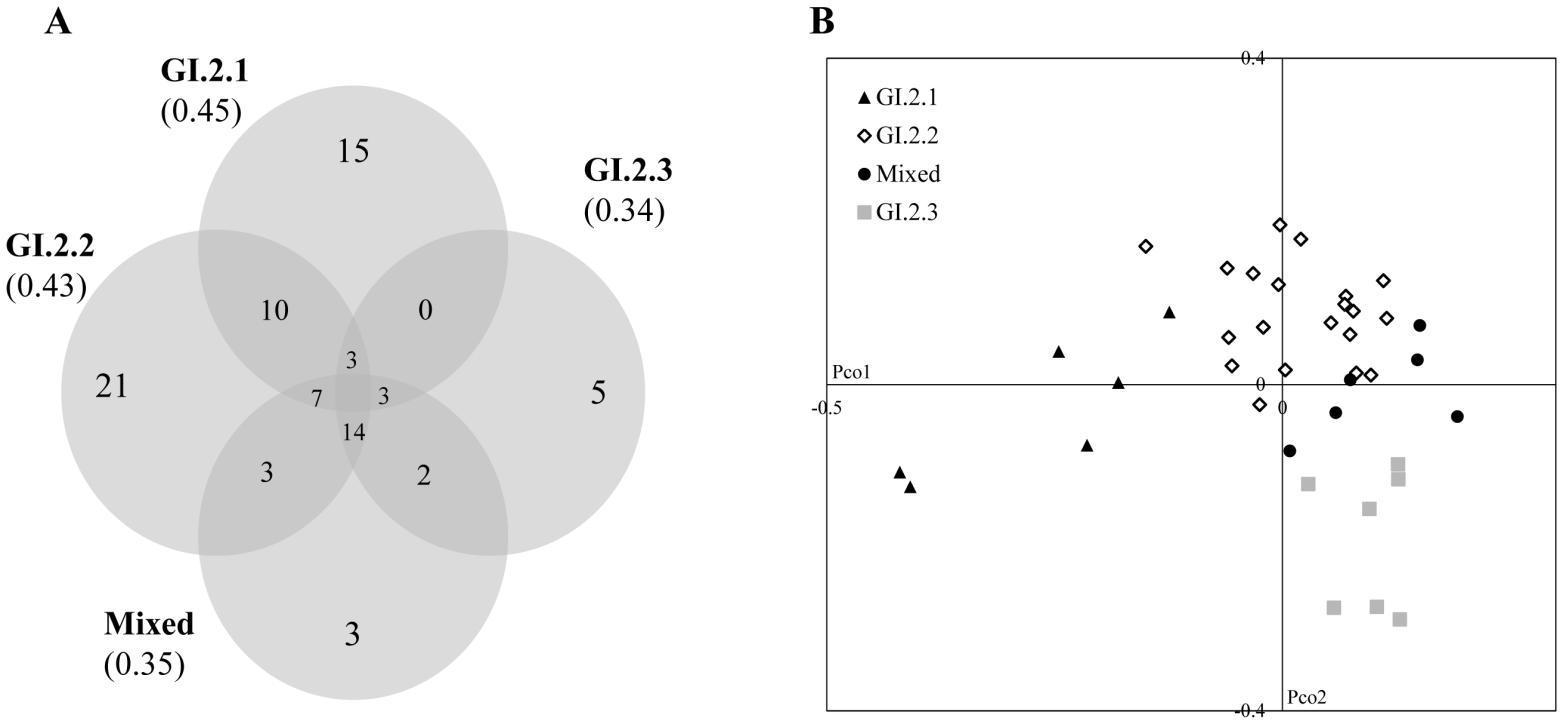
Venn diagrams and Principal Coordinate Analysis (PCoA) on the Spanish *C. annuum* groups established after structure analysis. A) Diagrams showing the number of unique alleles and shared alleles in the four groups defined by STRUCTURE in the Spanish *C. annuum* landraces. B) PCoA of the Spanish *C. annuum* accessions represented according to their memberships coefficients for *K* = 3.

Diversity based on the Nei's index was calculated for each STRUCTURE group at *K* = 3. The group GI.2.1 resulted somewhat the most variable (uHe = 0.45), followed by groups GI.2.2 (0.43), Mixed group (0.35) and GI.2.3 (0.34) (Fig. 4a). The detected genetic structure was more clearly reflected in the specific alleles. Hence, the group GI.2.2 could be easily differentiated from the other three due to the presence of 21 specific alleles at 14 loci. Fifteen unique alleles were found in the group GI.2.1 and only 3 and 5 alleles were detected in the Mixed group and GI.2.3, respectively. Common alleles were observed mainly between groups GI.2.1 and GI.2.2 and among GI.2.2/Mixed/GI.2.3 (Fig. 4a). Remarkably, 60% of the specific alleles present in group GI.2.1 were shared with non-Spanish *C. annuum* accessions, whereas 65%, 66.6% and 80% of the specific alleles displayed by GI.2.2, Mixed and GI.2.3 groups, respectively, resulted exclusive of *C. annuum* landraces from Spain. The groups established at *K* = 3 were visualized in the space through a principal coordinate analysis (Fig. 4b). The first coordinate explained a 12.07% of the variability and displayed the transition in fruit morphology from the group GI.2.1 (elongated short and round fruited accessions with a pungent allele in MAP1) to group GI.2.2 (mostly elongated), while the second dimension accounts for a 9.93% of the variance and exhibited the flux from group GI.2.2 to group GI.2.3 (triangular and blocky shaped forms with mainly non-pungent alleles), with the Mixed group arising between these two.

## Discussion

One hundred and two accessions from the *Capsicum* collection maintained at the Vegetable Germplasm Bank of Zaragoza (BGHZ) (Zaragoza, Spain) were evaluated with a set of microsatellite loci spread overall the pepper genome and mostly selected for their broad polymorphism in *Capsicum* spp [34], [37], [38], [46]. The level of transferability achieved by the SSR markers among the different species was high and mirror that experienced in other reports, particularly for EST-SSRs [34], [61], [62], since expressed sequences are more likely to be conserved between related species. Only a small number of genomic microsatellites generated null alleles for a few species, which agrees with results obtained by Nicolai et al. [48] in the *Capsicum* collection maintained at INRA (France). The rate of polymorphism for the entire collection was higher than that reported by other authors [37], [65], [66], [67] although slightly variable among *Capsicum* spp. Such variation might be interpreted to some extent as a bias of source markers, since the majority of primers were primarily designed for *C. annuum*. Additionally, the small number of accessions assessed for some of the species (for example *C. eximium*) as well as the larger diversity of some panels, concerning the geographical origin of accessions (for example *C. frutescens*), could also serve as plausible interpretations. In general, the PIC values represented more than 50% of polymorphism, which confirms that SSR markers used in this study were highly informative and that wide genetic variability has been accumulated within the collection.

The average number of alleles per locus was higher than those reported for germplasms sets from India or Japan [66], [68], but lower than those observed by Ibiza et al. [67] and Nicolai et al. [48] in other *Capsicum* collections. This might be explained because the latter reports surveyed larger samples than that of the current study. Nevertheless, a rough normalization of results according to the number of accessions per species suggests that our set is as diverse as those. Many private alleles were detected in the Spanish *Capsicum* collection, pointing to the existence of exotic accessions that could act as a reservoir for novel alleles for crop improvement. This was especially relevant for the *C. annuum* landraces from Spain, in which 37 unique alleles, i.e. not detected in the non-Spanish group, were identified. The presence of these alleles might be of adaptive significance, as a result of selection for adaptation to local constraints. Capture and preservation of these specific alleles and genotypes should be an important objective of any conservation strategy [69].

The observed heterozygosity in the entire collection was low and comparable to previous studies in *Capsicum* spp [48], [67], [70]. Such level of heterozygosity is expected due to the highly inbred nature of *Capsicum* populations in both domesticated and wild forms [71]. The genetic diversity, expressed as a measure of the Nei's unbiased gene diversity index, was similar to values reported in studies with larger diverse sets of *Capsicum* spp [48], [68], confirming that we are evaluating a sample of *Capsicum* types with considerable variability. The lowest diversity was found in *C. pubescens*, which also emerged as the less polymorphic group. This result agrees with the hypothesis of DeWitt and Bosland [72], who explained the narrowest genetic diversity of this species as a consequence of a founder effect during its domestication. Interestingly, *C. pubescens* showed the highest Ho within the domesticated species, despite the low mean number of alleles per locus, which might suggest a certain level of allogamy in this species. *C. frutescens* possessed the highest variation in terms of gene diversity (uHe), in accordance with results of Tam et al. [73]. In our case, the greatest variability obtained for this species could be also attributed to the diverse origins of the seven accessions that constitute the group, which come from Brazil to Japan and former Yugoslavia. Regarding *C. chinense* and *C. bacatum*, the diversity parameters were similar to *C. annuum* and comparable to those obtained in other reports [74], [75]. In this work, a relatively high level of genetic diversity was also observed for the cultivated *C. annuum*, as opposed to other self-pollinated crops. This fact was previously explained by Lefebvre et al. [31] as a consequence of the reproductive behaviour of domesticated pepper and the way in which domestication took place.

Multivariate and Bayesian analyses revealed that two accessions fell into different clusters from those of their supposed species. One likely explanation for these discrepancies is a misclassification of species based on morphological traits. Usually, classification of *Capsicum* spp based on morphology is carried out by investigating characters of flowers, leaves and fruits [43], but this method is sometimes ambiguous. This is especially true for *C. chinense* and *C. frutescens*, since the morphological characteristics of the flowers are similar in these two species. The structure of the dendrogram and PCoA were in good concordance with the taxonomical classifications previously reported for the genus *Capsicum* [11], [13], [71]. *C. chinense* and *C. frutescens*, clustered together in the NJ tree and they resolved as closely related to *C. annuum* in the space confined by the second coordinate in the PCoA analysis. This confirmed both species as sister taxa and outline the conventionally well-defined *C. annuum* complex [11], [76]. *C. cardenasii* and *C. eximium*, sometimes hypothesized as a single species, clustered together with a high bootstrap support confirming previous results [13], [67], [77], and together with *C. pubescens*, constituted a basal complex group characterised by purple flowers (*C. pubescens* complex) [8], [38], as demonstrated the PCoA output. Some inconsistences were observed regarding the classification of *C. chacoense*. The NJ tree suggested that *C. chacoense* accessions were more closely related to *C. bacatum*, although under a very weak bootstrapped node, coinciding with recent data from Ibiza et al. [67]. However, the ordination analysis grouped *C. chacoense* with the *C. pubescens* complex. *C. chacoense* was initially considered to be in either the *C. baccatum* or the *C. annuum* complex, since crosses between these species produce fertile hybrids and they share comparable morphotypes [8], [78]. However, other works resolved *C. chacoense* as a sister species to the purple-flowered group [11], [79]. Our results seem to indicate that *C. chacoense* might be closely related to both *C. baccatum* and *C. pubescens*, which is in agreement with earlier reports [48], [80] and support the hypothesis that *C. chacoense* may be the ancestor of the three major groups of *Capsicum* [12], [13]. The genetic relationship of *C. galapagoense* to the other *Capsicum* spp has been studied to a lesser extent and the majority of works resolved this species as a highly distinctive member of the *C. annuum* complex [11], [13]. Our data did not shed too much light on the placement of *C. galapagoense* within the *Capsicum* complexes, as the two analysed accessions were assorted in *C. chinense* and *C. baccatum* groups, which might be also attributed to morphological misclassifications.

An analysis of the entire collection with the STRUCTURE software, produced a most probable value of Δ*K* = 2, that clearly separated the *annuum* and non-*annuum* groups. Such explicit cut-off may well have resulted from the unbalanced size of the different *Capsicum* groups. Similarly, Rai et al. [66] observed a maximum Δ*K* value of 2, corresponding to *annuum* and non-*annuum* populations, in a neither equally distributed germplasm collection from India. Subsequent analysis of the non-*annuum* cluster reflected the separation of the majority of *Capsicum* species evaluated in this study according to their taxonomic group, in agreement with multivariate analyses Only *C. tovarii* was misplaced overall different analyses. The position of *C. tovarii* regarding to the other *Capsicum* spp have remained unclear [12], although some studies grouped it with the *C. baccatum* clade [13], [80]. However, our data suggested that *C. tovarii* might be included in the purple-flowered complex, constituted by *C. pubescens*, *C. eximium* and *C. cardenasii*. Nevertheless, considering that our results are based on only one accession, further investigations should be carried out to genetically position *C. tovarii* with finer resolution.

*C. annuum* is the most commonly cultivated species worldwide within the genus *Capsicum*. In Spain, several phenotypically diverse pepper landraces can still be found all over the country, due to their high quality and good acceptance in the national market. However, the origins and relationships among the landraces from different regions have remained unclear. Bearing in mind that pepper was likely introduce in Europe through Spain and that the Mediterranean region constitute an important secondary diversification center for *C. annuum* [17], we decide to investigate the genetic diversity, structure and putative diversification process in a sample of Spanish peppers, along with their relationship to a group of non-Spanish materials, also maintained at the BGHZ. The structure analysis firstly clustered the 51 cultivated *C. annuum* accessions into two principal genetic groups. A comprehensive examination of results suggested the grouping of genotypes according to fruit traits. This would explain why some Spanish accessions clustered with non-Spanish set, as all these individuals shared common fruit morphology, characterized by small and erect berries carrying an allele for pungency.

Independent model-based analysis within the Spanish group indicated that a Δ*K* value of 3 seemed to capture the major structure of the data, grouping the Spanish genotypes mostly according to the pepper fruit shape and size. In this manner, the group GI.2.1 was characterized by accessions with fruits that morphologically resemble traits attributed to the *C. annuum* ancestor; the wild bird pepper, such as the small size, red-colour, erect growth and pungency [23]. The other groups included on one hand mostly elongated fruited accessions carrying an allele for pungency (GI.2.2) and on the other hand large, triangular and blocky fruited accessions displaying a non-pungent allele in marker MAP1 (GI.2.3). Division of *C. annuum* genotypes according to the fruit shape has been previously reported for commercial cultivars and landraces [22], [32], [81], and earlier reports also pointed out clear separations among lines with small and pungent peppers, lines with elongated and pungent fruits and lines with large and blocky sweet peppers [23], [31], [38], [81]. Similarly, the large blocky and triangular types harboured lower degrees of genetic diversity, likely as a consequence of selection from a small gene pool and a bottleneck effect [24], [31], [48], [73]. A transition in membership coefficients between groups GI.2.2 and GI.2.3 was observed for some accessions, with a proportion of their genome into each group. These accessions, named as Mixed group, seemed to represent an intermediate genetic pool derived from proximal enlargement of elongate peppers as suggested by Tam et al. [73].

The inspection of subsequent values of *K* up to *K* = 5, revealed that the genotypes into group GI.2.3 (blocky and triangular types) remained tightly joined, but groups GI.2.1 and GI.2.2 were split into sub-groups. Hill et al. [82] observed a similar pattern in the genetic structure of a set of *C. annuum* cultivars for increased values of *K*, the Bell types persisting as a highly consistent group. In our case, differentiation between large triangular and blocky types was not possible. All these accessions are grown in close proximity in the North of Spain, so that gene flow between them might have occurred. That was consequent with observations by Portis et al. [83], who also found an indivisible group of triangular and blocky types, mostly from the North, within a collection of Italian pepper landraces. The most significant sub-division at *K* = 4 and *K* = 5 was the differentiation in cluster GI.2.2 of small blocky and small triangular fruited accessions from Northern Spain. These accessions might be derived from an ancestor in common with the cluster GI.2.1. Spain was the pepper's first point of arrival in Europe and it likely constituted a secondary diversification centre in the Mediterranean region [17], [84]. Such diversification might have occurred from a rather limited gene pool brought back from Central America in post-Columbian times. Part of the ancient populations could promptly migrate to the North of Spain, where they would suffer local selection and confinement to this geographical region leading to non-pungent, small blocky and triangular types. Indeed, some of the accessions, which comprised a specific cluster with a distinct genomic constitution at *K* = 5, retained one of the key traits that was left behind after domestication, as it is the erect peduncle of their flowers and fruits [23]. Large triangular and blocky fruited accessions grouped in cluster GI.2.3, also from the North, could be originated from these smaller types, throughout several years of farmer's selection towards an increase of fruit shape and a reduction in pungency. The remaining pepper accessions, mainly from Southern and Central Spain would diversify through different directions of artificial selection under the warmer and drier conditions of those regions. The PCoA analyses based on groupings at *K* = 3 supported the transition in morphologies suggested by the structure analysis, from peppers which resemble some wild-type characteristics to accessions selected for sweeter, larger and pendant fruits. This is in agreement with the overall trend during domestication and selection that has been reported in pepper [23], [73], [82], [85]. A clearer resolution of the relationships among *C. annuum* accessions from Spain will require of additional accessions and a larger amount of molecular markers in order to accurately investigate the impact of traditional farmer management in structuring genetic diversity and population dynamics of Spanish pepper landraces.

In the era of “Next Generation” DNA Sequencing (NGS) technologies, when thousands of high throughput molecular markers are already available and various *Capsicum* genomes have been sequenced [85], [86], [87], [88] yet rely on a set of microsatellite markers for evaluating genetic diversity could be interpreted as a limiting factor. Certainly, the use of such a small number of SSRs might have somehow hampered greater conclusions in the present work. However, microsatellites still remain very attractive for breeding purposes and they have proven superior power than other markers in resolving population structure [89]. Furthermore, SSRs can be assayed in any laboratory with minimum facilities and limited access to NGS tools. In the present study, the genotyping of the collection with a set of microsatellite markers, widely distributed across the twelve pepper chromosomes will serve to utilize, conserve and manage this pepper genetic pool in a more effective manner, widening their potential usage, and opening the way towards association genetics studies. A wide range of linkage disequilibrium (LD) across the *C. annuum* genome, which implies the feasible use of association mapping approaches, has been reported recently [90]. This level of LD could help to understand the consequences of breeding selection in *C. annuum* and the classification of Spanish landraces according to fruit traits as well as the transition in morphologies from ‘Chiltepin’-like peppers to the ‘Bell’-type forms. In any case, this is out of the scope of the current study and it should be addressed in future analyses. In conclusion, we can assert that the Spanish *Capsicum* germplasm collection held at the BGHZ represent a distinctive reservoir of genetic diversity, not only for the most widely grown *C. annuum* group, but also for other *Capsicum* species. Additionally, the BGHZ collection has proven to be a valuable resource to shed some light into the relationships among the divergent *Capsicum* species.

## Supporting Information

S1 Fig **Estimation of the number of groups based on output from the package STRUCTURE.** *K* values from 1 to 10 were explored by estimating the rate of change of the slope of the log likelihood curve (Δ*K*) calculated according to Evanno et al. [59] plotted against *K*. Plots for the analysis on the entire collection (A), on the non-*annuum* group (B), on the *C. annuum* group (C) and on the Spanish *C. annuum* accessions (D).
(TIF)

S2 Fig **Model based clustering for** ***C. annuum*** **accessions.** A) Structure bar plots based on 51 *C. annuum* accessions at *K* = 2, GI.1 = non-Spanish accessions, GI.2 = Spanish accessions. B) Spanish *C. annuum* population sub-structure for *K* = 4 and *K* = 5. Accessions are represented in columns and are ordered according to the probability of membership of every accession to each group, identified by colour.
(TIF)

S1 Table **Code, species, common name and origin of the** ***Capsicum*** **accessions selected from the BGHZ (Zaragoza, Spain).**
(XLSX)

S2 Table **Microsatellite markers evaluated for the genotyping of the** ***Capsicum*** **spp collection.**
(XLSX)

S3 Table **Total number of alleles and range of allele sizes detected in the entire collection, number of alleles and rate of polymorphism detected in each species.** The two numbers in brackets represent the number of unique alleles and the number of rare alleles, respectively. “Null” indicates the absence of amplification.
(XLSX)

S4 Table **PIC values of 39 SSR markers on the entire collection and in each species.** “Null” indicates the absence of amplification and “M” the absence of polymorphism.
(XLSX)
