# Supplementary figures and images for: New Insights into Capsicum spp Relatedness and the Diversification Process of Capsicum annuum in Spain
Source: PLoS One. 2014 Dec 29;9(12):e116276. doi: 10.1371/journal.pone.0116276 (PMC4278865; doi:10.1371/journal.pone.0116276)

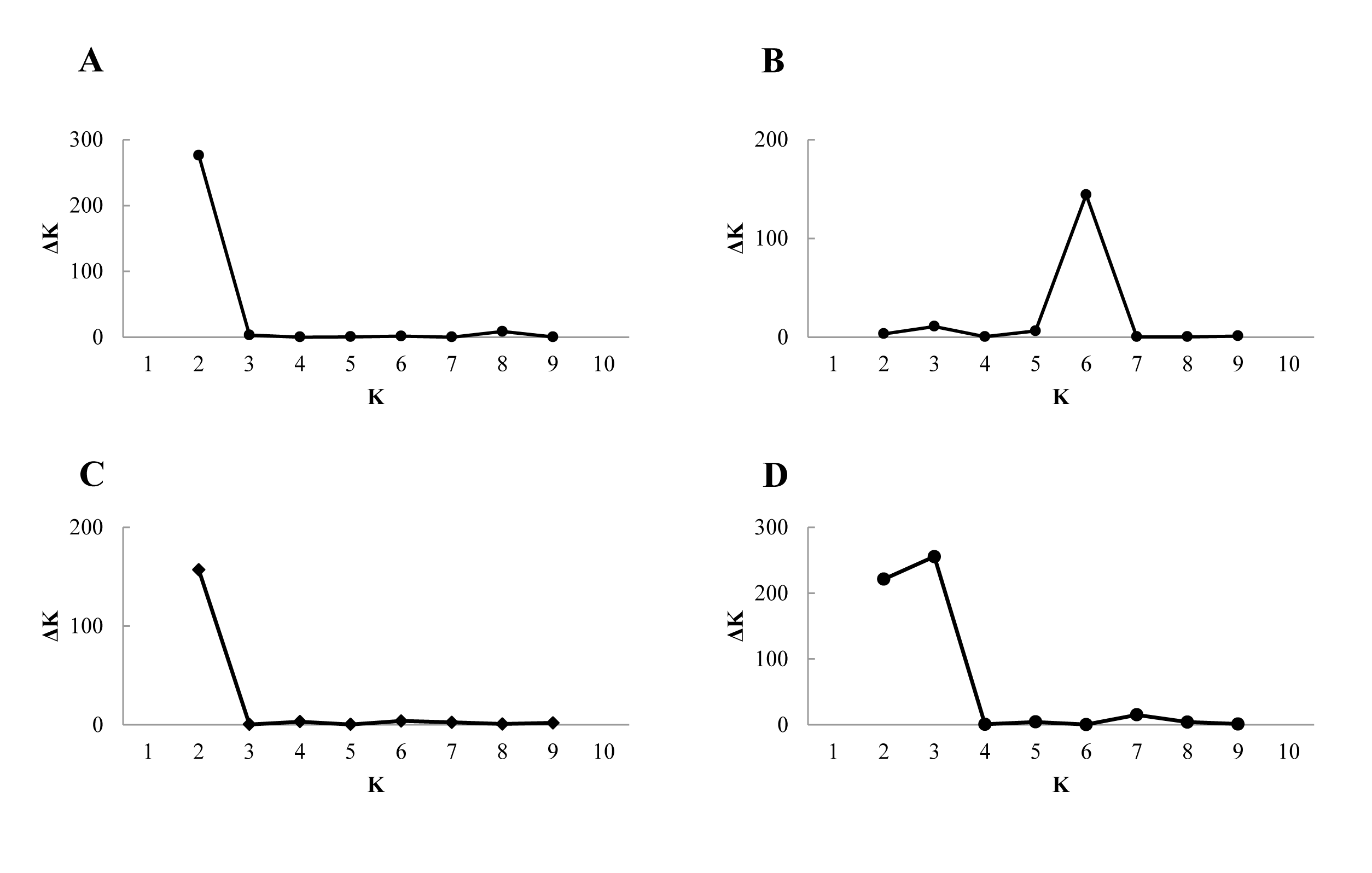

Supplement: S1 Fig — Estimation of the number of groups based on output from the package STRUCTURE. K values from 1 to 10 were explored by estimating the rate of change of the slope of the log likelihood curve (ΔK) calculated according to Evanno et al. [59] plotted against K. Plots for the analysis on the entire collection (A), on the non-annuum group (B), on the C. annuum group (C) and on the Spanish C. annuum accessions (D). (TIF) [file pone.0116276.s001.tif]

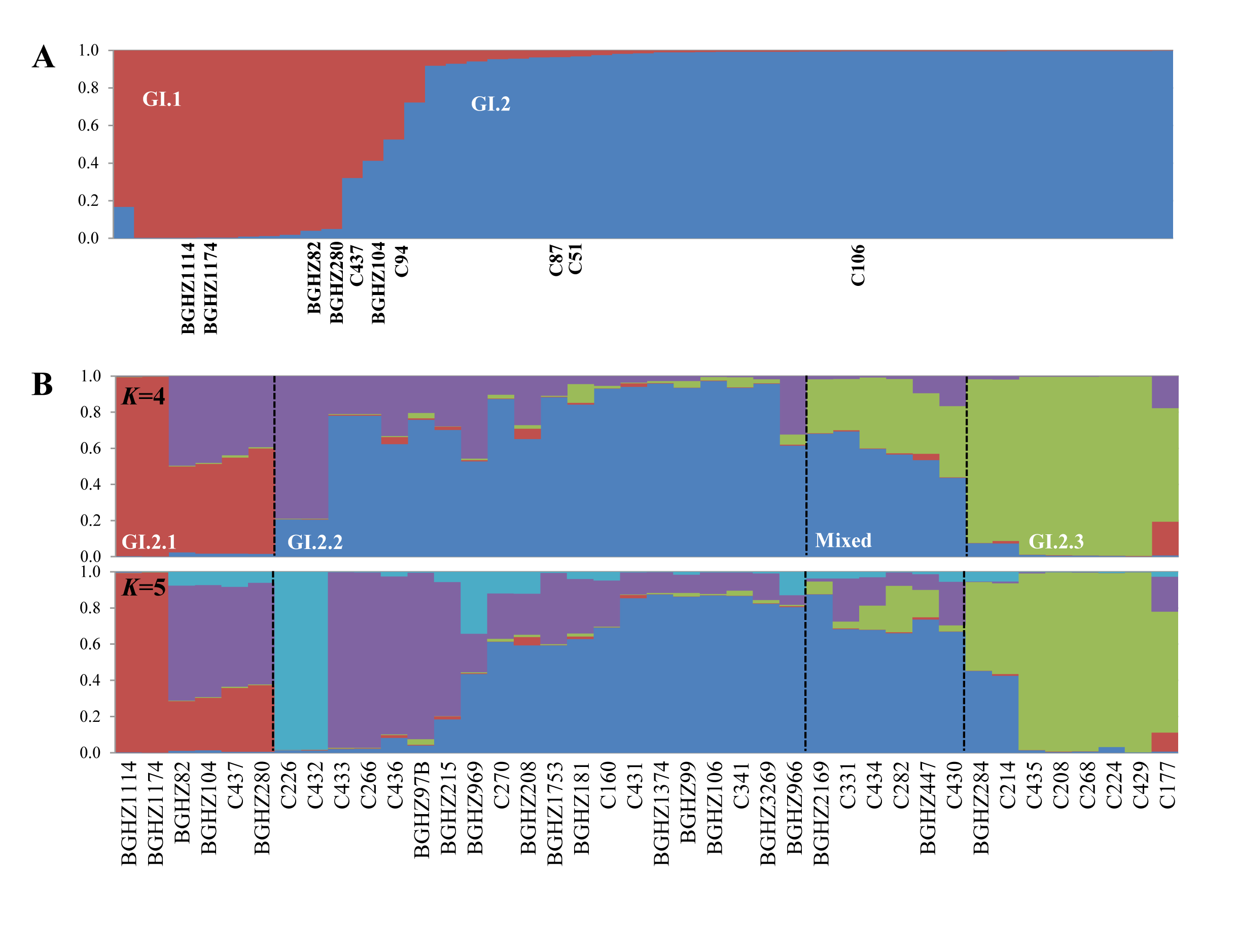

Supplement: S2 Fig — Model based clustering for C. annuum accessions. A) Structure bar plots based on 51 C. annuum accessions at K = 2, GI.1 = non-Spanish accessions, GI.2 = Spanish accessions. B) Spanish C. annuum population sub-structure for K = 4 and K = 5. Accessions are represented in columns and are ordered according to the probability of membership of every accession to each group, identified by colour. (TIF) [file pone.0116276.s002.tif]
